# Sample size requirements to detect the effect of a group of genetic variants in case-control studies

**DOI:** 10.1186/1742-7622-5-24

**Published:** 2008-12-03

**Authors:** Ramal Moonesinghe, Quanhe Yang, Muin J Khoury

**Affiliations:** 1 Office of Minority Health and Health Disparities, Centers for Disease Control and Prevention, Atlanta, Georgia, USA; 2 National Office of Public Health Genomics, Coordinating Center for Health Promotion, Centers for Disease Control and Prevention, Atlanta, Georgia, USA

## Abstract

**Background:**

Because common diseases are caused by complex interactions among many genetic variants along with environmental risk factors, very large sample sizes are usually needed to detect such effects in case-control studies. Nevertheless, many genetic variants act in well defined biologic systems or metabolic pathways. Therefore, a reasonable first step may be to detect the effect of a group of genetic variants before assessing specific variants.

**Methods:**

We present a simple method for determining approximate sample sizes required to detect the average joint effect of a group of genetic variants in a case-control study for multiplicative models.

**Results:**

For a range of reasonable numbers of genetic variants, the sample size requirements for the test statistic proposed here are generally not larger than those needed for assessing marginal effects of individual variants and actually decline with increasing number of genetic variants in many situations considered in the group.

**Conclusion:**

When a significant effect of the group of genetic variants is detected, subsequent multiple tests could be conducted to detect which individual genetic variants and their combinations are associated with disease risk. When testing for an effect size in a group of genetic variants, one can use our global test described in this paper, because the sample size required to detect an effect size in the group is comparatively small. Our method could be viewed as a screening tool for assessing groups of genetic variants involved in pathogenesis and etiology of common complex human diseases.

## Background

With the completion of the Human Genome Project and continuing advances in gene mapping and sequencing [1], there is an increasing interest in discovery and characterization of thousands of genetic variants as potential risk factors for common diseases of public health significance [2]. The search for genetic variants is currently hampered by numerous challenges, including the sheer number of genetic variants, the lack of replication of findings in many observational studies, and study design considerations (such as selection bias and confounding) [2-4]. Because the etiology of most common diseases such as cancer, heart disease and diabetes is due to complex genetic and environmental factors, a particular concern in the design of epidemiologic studies is the lack of statistical power to examine the joint effects and statistical interactions of several genetic variants, especially along with environmental risk factors [2]. For example, even if one considers that only 10 independent genetic variants are involved in a particular disease, and assuming simplistically a dichotomous classification of the susceptible genotype, this leads to more than a 1000 strata in which cases and controls can be distributed. With another 10 environmental dichotomous factors, we will have more than a million strata to assess. Note that the issue of multiple strata may be addressed by utilizing quantitative variables in the place of dichotomous variables where appropriate.

There have been several suggested methodologies to reduce the complex interactions of genetic and environmental effects, most notably multi-dimensionality reduction techniques, or MDR [5]. In the context of screening for the importance of a biologic system in the etiology of a specific disease, however, it is often helpful to have an *a priori *hypothesis for the genetic effects that belong to a certain biologic pathway. For example, in studying the etiology of venous thrombosis, researchers are examining the effects of genetic variants involved in the coagulation pathway [6]. Also, in studying the etiology of neural tube defects (NTD), because of the protective effects of dietary folates, researchers are examining the relationship between genetic variants involved in folate metabolism and the risk of NTD [7].

In this paper, we present a simple method for assessing the overall effect of a group of genetic variants in the context of case-control studies. Although *post hoc *tests have to be conducted to assess joint effects of combinations of specific genetic variants, our method enables detection of the average effect of the group of genetic variants with a reasonable sample size; it can thus be used as a screening approach for further study.

### Analysis

Mckeown-Eyssen and Thomas [8] explored the relationship between exposure and the differences in case-control means when the distribution of exposure is continuous. They derived sample size equations for studies with a continuous exposure, which allow the investigator to specify the strength of the relationship between disease and exposure in terms of relative risk. Given the joint distribution of exposure for controls, Rao [9] derived the joint distribution for the exposure of cases by dividing the product of the joint distribution of exposure for controls and the risk function by the sum of this product over all the possible values that the exposure variable can assume. We used this method to derive sample size formulas given a joint distribution of k-genetic variants for multiplicative and additive models. The result of our investigations of multiplicative models is presented below.

Suppose that the population at risk is exposed to a level X_i _of the i^th ^genetic variant (X_i _can assume only 1 or 0 depending on the presence or absence of the i^th ^genetic variant). Let G_1_, G_2_,..., G_k_, and R_1_, R_2_,..., R_k_, be the prevalence and the relative risks for the k-genetic variants, which are assumed to be known. Also, let U_1_, U_2_,..., U_k_, denote the exposure variables (U_i _can assume only 0 or 1) among cases for the k-genetic variants. Let $\bar{X}$ = (${\bar{\text{X}}}_{1},{\bar{\text{X}}}_{2},...,{\bar{\text{X}}}_{\text{k}}$) be the vector of sample means for controls for the k-genetic variants (assuming the probability of disease is small) and $\bar{U}$ = (${\bar{\text{U}}}_{1},{\bar{\text{U}}}_{2},...,{\bar{\text{U}}}_{\text{k}}$) be the corresponding vector of sample means for cases. We assume equal sample sizes for cases and controls and a multiplicative risk model. The test for a difference in mean exposure levels of the group of k-genetic variants is given by:

H_0_: R_1 _= R_2 _= ... = R_k _= 1 versus H_1_: at least one R_i _≠ 1.

For large sample sizes, the simultaneous test for difference in prevalence between cases and controls is:

$${\text{Reject H}}_{0}\text{ if T}=\frac{n}{2}(\bar{X}-\bar{U})\Sigma^{-1}{\left(\bar{X}-\bar{U}\right)}^{T}\geq \chi_{k,\alpha }^{2},$$

where $\chi_{k,\alpha }^{2}$ is the 100(1-α)% probability point of the chi-square distribution with k degrees of freedom and Σ is the variance covariance matrices for $\bar{X}$ (or $\bar{U}$) under the null hypothesis (Appendix A1). Under the alternative hypothesis H_1_, using a conservative simplification due to Lachin [10], the distribution of the test statistic has a non-central chi-squared distribution with k degrees of freedom and non-centrality parameter δ (i.e. $\chi_{k}^{2}(\delta )$), where

$$\delta =\frac{n}{2}(G-G^{*})\Sigma^{-1}{\left(G-G^{*}\right)}^{T},$$

and

**G **= (G_1_, G_2_,..., G_k_) and G* = (G_1 _*, G_2 _*,..., Gk*) are the vectors of prevalence of the k genetic variants for controls and cases, respectively. If the test is required to have a specified power (1-β), δ is calculated as the solution to the equation $\chi_{k,(1-\beta )}^{2}(\delta )=\chi_{k,\alpha }^{2}$. The sample size required to detect a difference in mean exposure for the group of k-genetic variants with (1-β) power at α level significance is given by:

(1) $$n=\frac{2\delta }{(G-G^{*})\Sigma^{-1}{\left(G-G^{*}\right)}^{T}}$$

If the null hypothesis, H_0_, is rejected, one can conduct subsequent multiple tests to detect which R_i_s are significantly different from 1 or test subsets of R_i_s using the same test statistic given above. However, the level of significance of each test has to be adjusted based on the number of multiple tests conducted.

## Results

**Figure 1:**
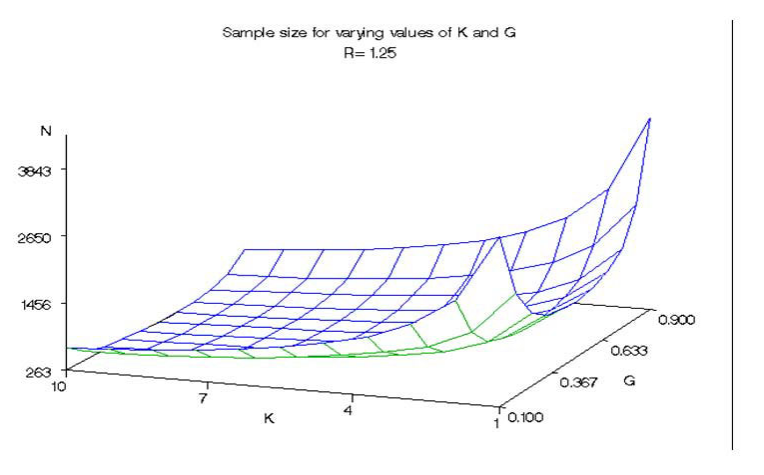
**Sample size for varying values of K and G (R = 1.25)**. Approximate sample sizes required to achieve 80% power at 5% significance level in detecting the difference in mean exposure between cases and controls due to a hypothetical group of k identical genetic variants with a risk ratio of 1.25.

**Figure 2:**
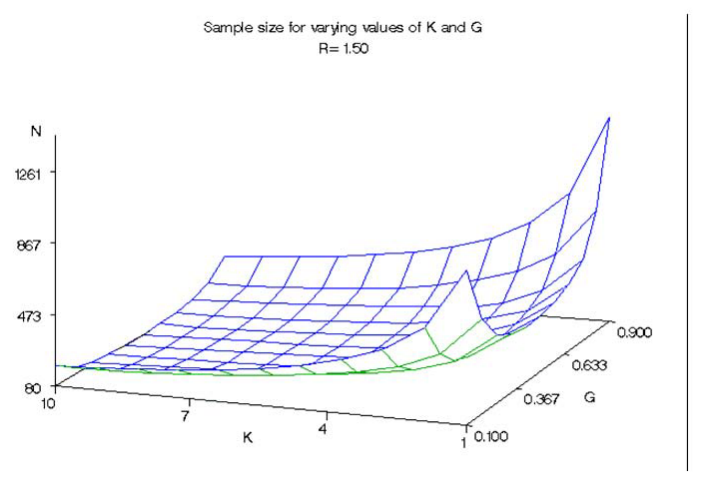
**Sample size for varying values of K and G (R = 1.50)**. Approximate sample sizes required to achieve 80% power at 5% significance level in detecting the difference in mean exposure between cases and controls due to a hypothetical group of k identical genetic variants with a risk ratio of 1.50.

**Figure 3:**
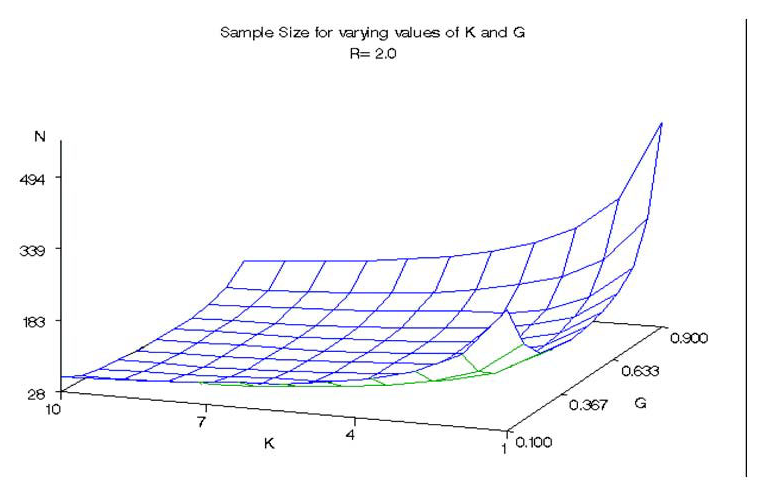
**Sample size for varying values of K and G (R = 2.0)**. Approximate sample sizes required to achieve 80% power at 5% significance level in detecting the difference in mean exposure between cases and controls due to a hypothetical group of k identical genetic variants with a risk ratio of 2.0.

We calculated the sample size required to detect a hypothetical group of k identical genetic variants (all loci are equivalent having equal effects and are independent). Figures 1, 2, and 3 give the approximate sample size (number of cases in 1:1 design) required to achieve 80% power at 5% significance level for detecting mean exposure due to a hypothetical group of k identical genetic variants when the prevalence varies from 0.1 to 0.9 and k varies from 1 to 10. Figure 1 corresponds to a risk ratio of 1.25, while figures 2 and 3 assume risk ratios of 1.5 and 2.0, respectively.

Overall, the sample size requirement declined with increasing values of k. For example, compared with the sample size requirement for k = 1 the sample size requirement for k = 10 declined by approximately 79% on average for all prevalence and risk ratios studied. Prevalences of 0.9 and 0.1 corresponded to the largest sample sizes for all the risk ratios and numbers of genetic variants in the group. There was little difference between sample size requirements for prevalence ranges between 0.3 and 0.6 for large values of k for the given risk ratios. When k is greater than 4 and R = 2.0, the difference in required sample size for the range of prevalence from 0.3 to 0.6 was less than 6 observations. Indicative of this result, the surfaces shown in all three figures have a relatively flat bottom for k greater than 4 and for the range of prevalence from 0.3 to 0.6. As expected, the sample size requirement declined with increasing R. A theoretical explanation of these results is given below.

Let G be the prevalence in the population of the genetic variants in the hypothetical group of k identical genetic variants and G* be the prevalence in cases. We assume independent genetic variants. The denominator in (1) is then given by

(2) $$\begin{array}{ccc} \frac{\text{k}{\left(\text{G}-{\text{G}}^{*}\right)}^{2}}{\bar{\text{G}}(1-\bar{\text{G}})} & \text{where} & \bar{\text{G}}=0.5*(\text{G}+{\text{G}}^{*}). \end{array}$$

Let n_k _be the sample size requirement corresponding to the group of k genetic variants. Then from (1) and (2),

$$\frac{{\text{n}}_{\text{k}}}{{\text{n}}_{1}}=\frac{1}{\text{k}}\frac{\delta_{\text{k}}}{\delta_{1}}$$

where δ_k _is the non-centrality parameter of a chi-squared distribution with k degrees of freedom. This result shows that for k = 10,

$$\frac{{\text{n}}_{10}}{{\text{n}}_{1}}=\frac{1}{10}\frac{\delta_{10}}{\delta_{1}}=0.207$$

and for any given G and R in the hypothetical group of k identical variants, the sample size requirement for k = 10 declined by 79.3% compared to the sample size requirement for k = 1. In a similar manner, one can show that

$$\frac{{\text{n}}_{\text{k}+1}}{{\text{n}}_{\text{k}}}=\frac{\text{k}}{\text{k}+1}\frac{\delta_{\text{k}+1}}{\delta_{\text{k}}}$$

The difference between δ_k+1 _and δ_k _declines with increasing k and $\frac{\text{k}}{\text{k}+1}$ approaches 1 for large values of k; hence, the successive difference between sample size requirements declines with increasing values of k.

### Example

Yang [11] provided an example of variants at five genes that have been associated with the risk of colorectal cancer. As shown in Table 1, the prevalence of the variants ranged from 4.0% to 60% and the odds ratios ranged from 1.35 to 2.67. Although these genetic variants do not necessarily belong to one biologic system, we use them here only to illustrate the method. Table 2 gives the sample size requirements (number of cases in 1:1 case-control design) to detect the difference in mean exposure of the group of genetic variants between cases and controls when the group consists of different combinations of genetic variants given in Table 1.

**Table 1:** Prevalence and odds ratios of five genetic variants for colorectal cancer susceptibility.

Genetic variants	Risk group	Genotype prevalence%	Odds ratio
*HRAS1 *(1)	Rare allele vs. others	4.0	2.67
*GSTT1 *(2)	Null vs. others	37.6	1.37
*TNF-α *(3)	α2 allele vs. others	39.2	2.02
*NAT2 *[imputed from phenotype] (4)	Fast acetylation vs. others	[60.3]	1.68
*MTHFR *(5)	Wild-type vs. variant (C677T)	42.3	1.35

(*HRAS1 *= c-Ha-ras1 proto-oncogene; *GSTT1 *= glutathione S-transferase theta 1; *TNF-α *= tumor necrosis factor alpha-chain; *NAT2 *= N-acetyl transferase-2 gene; *MTHFR *= 5,10-methylenetetrahydrofolate reductase gene.)

**Table 2:** Sample size requirement to detect mean exposure between cases and controls for some combinations of genetic variants given in Table 1 assuming multiplicative risk

Genetic variants	Sample size	Genetic Variant	Sample size
(1) *HRAS1*	283	(4)+(5)	236
(2) *GSTT1*	656	(1)+(2)+(3)	109
(3) *TNF-α*	130	(1)+(2)+(4)	158
(4) *NAT2*	265	(1)+(2)+(5)	215
(5) *MTHFR*	705	(1)+(3)+(4)	93
(1)+(2)	243	(1)+(3)+(5)	110
(1)+(3)	110	(2)+(3)+(4)	107
(1)+(4)	168	(2)+(3)+(5)	181
(1)+(5)	248	(3)+(4)+(5)	108
(2)+(3)	134	(1)+(2)+(3)+(4)	92
(2)+(4)	232	(1)+(2)+(3)+(5)	107
(2)+(5)	417	(2)+(3)+(4)+(5)	106
(3)+(4)	107	(1)+(2)+(3)+(4)+(5)	91
(3)+(5)	135		

GSTT1 and MTHFR have the smallest odds ratios (1.37 and 1.35 respectively) in Table 1 and the largest sample size requirements (656 and 705 respectively). The higher sample size for MTHFR reflects the small difference (0.02) in R, even though the prevalence for MTHFR is greater than for GSTT1 (0.423 versus 0.376). This shows that when prevalence is closer to 0.5, the sample size requirement is more sensitive to the differences in R. The smallest sample size (130) corresponds to TNF-α, which has an odds ratio of 2.02 and a prevalence of 0.392. The largest odds ratio, 2.67, for HARS1 corresponds to a larger sample size due to the very low prevalence (0.04).

These results for individual genetic variants seem to carry over to the group of genetic variants. For example, the sample size requirement to detect a group of two genetic variants out of the five given in Table 1, the combination GSTT1 and MTHFR, corresponds to the largest sample size (417), and the combination TNF-α and NAT2, which have odds ratios of 2.02 and 1.68, respectively, corresponds to the smallest sample size (107). For a group of three genetic variants, the combination HRAS1, GSTT1 and MTHFR corresponds to the largest sample size requirement (215). These are the three genetic variants that have the largest sample size requirements when considered individually. Overall, as seen before, the sample size requirement declined with the increase in the number of genetic variants in the group. The sample size requirement for all the genetic variants given in Table 1 is 91.

## Conclusion

We have presented a simple method for estimating the sample size for case-control studies required to detect a group of genetic variants using multiplicative models. We have also used the same approach for additive risk models; however, we could not show the asymptotic normality of the joint distribution of exposure for cases (Appendix A2).

In the multiplicative model, when the genetic variants are found to be jointly significant, subsequent multiple tests could be conducted to detect which R_i_s are significantly different from 1. For example, if the null hypothesis is rejected for a group of five genetic variants, and R_1_, R_2 _and R_5 _are significantly different from 1, we can conclude that the joint effect of G_1_, G_2 _and G_5 _is significantly different between cases and controls.

Consider k hypothesis tests. Under the null hypothesis using the Bonferroni inequality, the probability that at least one of the k tests is significant at level α_0 _is less than or equal to α_0_k. In order to maintain an overall level of significance α, we would use the significance level α_0 _= α/k for each of the k separate tests of significance. Several less conservative adjustments for multiple tests of significance have been proposed, such as the procedure of Holm [12] and Hochberg [13]. All of these procedures conduct the multiple tests by ordering the test statistics from largest to smallest and then using less restrictive significance levels to the second, third, and so on, test conducted. When any one test is not significant, the procedure stops and all further tests are also declared non-significant. Benjamin [14] suggested that the False Discovery Rate (FDR) may be the appropriate error rate to control in many applied multiple testing problems. The FDR is the expected proportion of erroneous rejections among all rejections. A simple procedure was given there as an FDR controlling procedure for independent test statistics and was shown to be much more powerful than comparable procedures that control the traditional family-wise-error-rate (the probability of erroneously rejecting even one of the true null hypotheses).

One could have conducted a simultaneous test of the k-parameter joint null hypothesis using multiple tests discussed above as an alternative approach to our test. However, all these tests are conservative compared to the multivariate test presented here. On the other hand, multiple comparison tests could be applied in instances in which the k-statistic vector is not normally distributed, making these tests suitable for the additive model given in the Appendix A2.

Garcia-Closas [15] evaluated the influence of common genetic variation in the NER pathway on bladder cancer risk by analyzing 22 single nucleotide polymorphisms (SNP) in seven NER genes (XPC, RAD23B, ERCC1, ERCC2, ERCC4, ERCC5, and ERCC6). They estimated odds ratios for each individual polymorphism using logistic regression. They then performed a global test for the association between genetic variations in NER pathway as a whole based on the maximum of trend statistics of all the individual polymorphisms. The P-value for the global test was computed by the permutation method described in Westfall [16]. They found significant associations with SNPs in four of the seven NER genes. They used 1150 cases and an almost equal number of controls. The p-value for the global test for pathway effects was 0.04. Their minor allele frequencies ranged from 0.01 to 0.33 and the odds ratios ranged from 0.8 to 1.4 with an average odds ratio of 1.2. If the odds ratios and SNP frequencies were known (assuming an average odds ratio of 1.2 and a dominant model), the sample size required to achieve 80% power at the 5% level of significance in detecting the overall effect of 22 SNPs using our method is 212 cases. In situations in which we find that none of the genetic variants were significant, the method described in this paper could have reduced the cost of the experiment by first screening the group of genetic variants for overall significance.

The results obtained here can be easily extended to a group of k genetic variants and l environmental factors, when the exposure to the i^th ^environmental factor can be specified as E_i _= 1 (present) or E_i _= 0 (absent) and the E_i_s are independent among themselves and are independent of the genetic variants.

Our approach is limited by its inability to look at higher order interactions and the assumption of independence between all loci. Covariance terms in the variance-covariance matrix could increase the sample size to detect the group of genetic variants. It is possible that we may not detect individual effects, but there may be joint effects due to interactions. Our method cannot detect these interactions. Our sample size is constrained by our assumption of normal approximation to binomial distribution. Another limitation is the assumption of multiplicative effects of genetic variants. True biologic interactions could be more complex with epistasis and/or other genetic phenomena; furthermore, joint genetic effects and gene-environment interactions on risk may be neither additive nor multiplicative. Unfortunately, for statistical modeling, epidemiologic analyses have had to deal with multiplicative or additive models. The rare disease assumption in case-control studies has been discussed in many papers [17,18]. Generally, since most diseases are infrequent, ORs are good estimators of relative risks under this "rare disease assumption". For a disease with a frequency of 10%, which is high, the difference between OR and RR is still only 10%. The only requirement in our genetic model is the ability to express exposure due to genotype as 1 (presence of genotype) or 0 (absence of genotype). Therefore, either dominant or recessive models can be used in our analysis.

A non-parametric approach to this problem is the method of Multidimensionality Reduction (MDR), introduced by Ritchie [5] as a method of reducing the dimensionality of multilocus information to improve the identification of polymorphism combinations associated with disease risk. This data reduction approach seeks to identify combinations of multilocus genotypes and discrete environmental factors that are associated either with high risk of disease or low risk of disease, and defines a single variable that can be divided into high-risk and low-risk combinations. When it was applied to a sporadic breast cancer case-control data set, in the absence of statistically significant independent main effects, MDR identified a statistically significant higher-order interaction among four polymorphisms from three different estrogen-metabolism genes. Limitations of MDR include its applicability only to case-control studies that are balanced, and the difficulty in interpreting MDR models. Three different strategies for improving the power of MDR to detect epistasis in imbalanced datasets have been evaluated in a recent paper[19].

Another recent approach that holds great promise is logic regression, introduced by Ruczinski [20] as a tool to detect interactions between binary predictors that are associated with a response variable. Logic regression is an adaptive regression methodology that attempts to construct predictors as Boolean combinations of binary covariates. According to the authors, logic regression is the only methodology that searches for Boolean combinations of predictors in the entire space of such combinations, while being completely embedded in a regression framework, where the quality of the model is determined by the respective objective functions of the regression class.

Suppose there are k genetic variants in a group of genetic variants and only r of them are associated with the disease. The prevalence of each of (k-r) genetic variants that are not associated with the disease (relative risk of each genetic variant is equal to 1) is identical for cases and controls. Therefore, from equation (1), the sample size required to detect the k genetic variants is identical to the sample size required to detect the r genetic variants associated with the disease. Since our sample size is a function of the squares of the difference between prevalence of genetic variants in cases and controls, our method is valid even when we have a combination of positively and negatively associated genetic variants.

One advantage of our method is the simultaneous test of difference of mean exposure instead of multiple testing. Thus, for a range of reasonable numbers of genetic variants, the sample size requirement declines with the increasing number of genetic variants. It is possible that the sample size required to detect a group of genetic variants could increase when adding a genetic variant to the group. However, the sample size required to detect the group with this genetic variant is still less than the sample size required to detect the genetic variant alone or to detect a subset of the genetic variants containing this genetic variant. When testing for an effect size in a group of genetic variants, one can use the global test described in this paper as a screening tool, because the sample size required to detect an effect size in the group is comparatively small. Note that we are comparing the ability to detect at least one of many genetic variants (global test) with the power to detect just one, which are different null hypotheses. If the global test is non-significant, testing for individual genetic variants that require a large sample size is not necessary.

More methodological work is needed in this area to detect joint effects of multiple genetic variants. Our method could be viewed as a screening tool for assessing groups of genetic variants involved in pathogenesis and etiology of common complex human diseases.

## Competing interests

The authors declare that they have no competing interests.

## Authors' contributions

RM led statistical analysis and drafted parts of manuscript. QY contributed to statistical design. MK designed, led overall study and drafted parts of manuscript. All authors read and approved the manuscript.

## Appendix A1

Let f_0_(X_1_, X_2_,..., X_k_) be the joint probability density function among controls and f_1_(X_1_, X_2_,..., X_k_) be the joint probability density function among cases. If $\bar{\text{D}}$ denotes controls and D denotes the cases, then

f_0_(X_1_, X_2_,..., X_k_) = Pr [(X_1_, X_2_,..., X_k_)|$\bar{\text{D}}$] and f_1_(X_1_, X_2_,..., X_k_) = Pr [(X_1_, X_2_,..., X_k_)| D]

The probability density function of the exposure variables in the population at risk becomes:

f(X_1_, X_2_,..., X_k_) = f_0_(X_1_, X_2_,..., X_k_)Pr($\bar{\text{D}}$) + f_1_(X_1_, X_2_,..., X_k_)Pr(D)

Assuming the probability of disease is small, we can approximate the distribution of the exposures among the controls by that present among the general population.

f(X_1_, X_2_,..., X_k_) ≈ f_0_(X_1_, X_2_,..., X_k_).

Assuming that the exposure variables corresponding to the k-genetic variants are independent, the joint distribution of the k exposure variables is given by

(A) $$\text{f}({\text{X}}_{1},{\text{X}}_{2},...,{\text{X}}_{\text{k}})={\text{G}}_{1}^{{\text{X}}_{1}}{\left(1-{\text{G}}_{1}\right)}^{\left(1-{\text{X}}_{1}\right)}...{\text{G}}_{\text{k}}^{{\text{X}}_{\text{k}}}(1-{\text{G}}_{\text{k}}^{{\text{X}}_{\text{k}}})=\prod_{\text{i}=1}^{\text{k}}{\text{G}}_{\text{i}}^{{\text{X}}_{\text{i}}}{\left(1-{\text{G}}_{\text{i}}\right)}^{\left(1-{\text{X}}_{\text{i}}\right)}$$

Consider the multiplicative risk model:

(B) $$\text{R}({\text{X}}_{1},{\text{X}}_{2},...,{\text{X}}_{\text{k}})={\text{IR}}_{1}^{{\text{X}}_{1}}...{\text{R}}_{\text{k}}^{{\text{X}}_{\text{k}}}=\text{I}\left[\prod_{\text{i}=1}^{\text{k}}{\text{R}}_{\text{i}}^{{\text{X}}_{\text{i}}}\right],$$

where I is the background risk. The average rate of disease in the population at risk is given by

$$\text{M}=\sum_{{\text{X}}_{1},{\text{X}}_{2},...,{\text{X}}_{\text{k}}}\text{R}({\text{X}}_{1},{\text{X}}_{2},...,{\text{X}}_{\text{k}})\text{f}({\text{X}}_{1},{\text{X}}_{2},...,{\text{X}}_{\text{k}})$$

The summation is over all the possible values each X_i _can assume (0 and 1).

Using (A) and (B), it can be shown that

$$\begin{array}{c} \text{M}=\text{I}\prod_{\text{i}=1}^{\text{k}}\left[\sum_{{\text{X}}_{\text{i}}=0}^{1}{\text{R}}_{\text{i}}^{{\text{X}}_{\text{i}}}{\text{G}}_{\text{i}}^{{\text{X}}_{\text{i}}}{\left(1-{\text{G}}_{\text{i}}\right)}^{\left(1-{\text{X}}_{\text{i}}\right)}\right]\\ =\text{I}\prod_{\text{i}=1}^{\text{k}}\left[{\text{R}}_{\text{i}}{\text{G}}_{\text{i}}+(1-{\text{G}}_{\text{i}})\right]. \end{array}$$

Yang [11] defined M as the lifetime risk in the population as a whole of a common disease involving k-genetic variants for multiplicative models.

If U_1_, U_2_,..., U_k_, denote the exposure variables (U_i _can assume only 0 or 1) among cases for the k-genetic variants, their joint probability density function is given by the product of the risk function and the probability density function of the exposure variables in the controls divided by M. Lui [21] derived the joint probability density function for exposure variables in cases using this approach when exposure variables have a multivariate normal distribution. The distribution g of U_1_, U_2_,..., U_k _is given by

(C) $$\begin{array}{c} \text{g}({\text{U}}_{1},{\text{U}}_{2},...,{\text{U}}_{\text{k}})=\{\text{f}({\text{U}}_{1},{\text{U}}_{2},...,{\text{U}}_{\text{k}})\text{R}({\text{U}}_{1},{\text{U}}_{2},...,{\text{U}}_{\text{k}})\}/\text{M}\\ =\text{I}\prod_{\text{i}=1}^{\text{k}}{\text{G}}_{\text{i}}^{{\text{U}}_{\text{i}}}{\left(1-{\text{G}}_{\text{i}}\right)}^{\left(1-{\text{U}}_{\text{i}}\right)}\prod_{\text{i}=1}^{\text{k}}{\text{R}}_{\text{i}}^{{\text{U}}_{\text{i}}}/\text{M}\\ =\prod_{\text{i}=1}^{\text{k}}\frac{{\left({\text{R}}_{\text{i}}{\text{G}}_{\text{i}}\right)}^{{\text{U}}_{\text{i}}}{\left(1-{\text{G}}_{\text{i}}\right)}^{\left(1-{\text{U}}_{\text{i}}\right)}}{\left[{\text{R}}_{\text{i}}{\text{G}}_{\text{i}}+(1-{\text{G}}_{\text{i}})\right]}\\ =\prod_{\text{i}=1}^{\text{k}}({\text{G}}_{\text{i}}^{*}{)}^{{\text{U}}_{\text{i}}}(1-{\text{G}}_{\text{i}}^{*}{)}^{\left(1-{\text{U}}_{\text{i}}\right)} \end{array}$$

where ${\text{G}}_{\text{i}}^{*}=\frac{{\text{R}}_{\text{i}}{\text{G}}_{\text{i}}}{{\text{R}}_{\text{i}}{\text{G}}_{\text{i}}+(1-{\text{G}}_{\text{i}})}$.

A comparison of (A) with (C) shows that the joint distribution of exposure among cases has the same form as that of controls; however, they have different parameters for prevalence of the genetic variants and the assumption of independence of exposure variables for controls results in the independence of exposure variables for cases. The prevalence of the i^th ^genetic variant among cases is given by ${\text{G}}_{\text{i}}^{*}$.

The mean exposure levels of the k-genetic variants for controls is given by G_i_, for i = 1, 2,..., k. Similarly the mean exposure levels of the k-genetic variants for cases is given by ${\text{G}}_{\text{i}}^{*}$ for i = 1, 2,..., k. The test for a difference in mean exposure levels of the group of k-genetic variants is given by:

H_0_: R_1 _= R_2 _= ... = R_k _= 1 versus H_1_: at least one R_i _≠ 1.

This test is identical to the test:

H_0_:G_i _= ${\text{G}}_{\text{i}}^{*}$ for i = 1, 2,.., k, versus H_1_:G_i _≠ ${\text{G}}_{\text{i}}^{*}$ for at least one i (i = 1, 2,.., k).

We assume equal sample sizes for cases and controls. For a large sample size n (the sample size for controls or cases), the variance-covariance matrices of $\bar{X}$ and $\bar{U}$ are given by $\frac{\text{1}}{\text{n}}\Sigma_{1}$ and $\frac{\text{1}}{\text{n}}\Sigma_{2}$ respectively where

$$\begin{array}{l} {\left(\Sigma_{1}\right)}_{\text{i},\text{i}}={\text{G}}_{\text{i}}(1-{\text{G}}_{\text{i}})\text{ and }{\left(\Sigma_{1}\right)}_{\text{i},\text{j}}=0\text{ for i}\neq \text{j},\text{ and}\\ {\left(\Sigma_{2}\right)}_{\text{i},\text{i}}={\text{G}}_{\text{i}}^{*}(1-{\text{G}}_{\text{i}}^{*})\text{ and }{\left(\Sigma_{2}\right)}_{\text{i},\text{j}}=0\text{ for i}\neq \text{j} \end{array}$$

Under the null hypothesis, the variance covariance matrices for $\bar{X}$ and $\bar{U}$ are equal and may be written

$${\left(\Sigma \right)}_{\text{i},\text{i}}={\bar{\text{G}}}_{\text{i}}(1-{\bar{\text{G}}}_{\text{i}})\text{ and }{\left(\Sigma \right)}_{\text{i},\text{j}}=0\text{ for i}\neq {\text{j where }\bar{\text{G}}}_{\text{i}}=0.5({\text{G}}_{\text{i}}+{\text{G}}_{\text{i}}^{*}).$$

For large sample sizes, the simultaneous test for difference in prevalence between cases and controls is:

$${\text{Reject H}}_{0}\text{ if T}=\frac{n}{2}(\bar{X}-\bar{U})\Sigma^{-1}{\left(\bar{X}-\bar{U}\right)}^{T}\geq \chi_{k,\alpha }^{2},$$

where $\chi_{k,\alpha }^{2}$ is the 100(1-α)% probability point of the chi-square distribution with k degrees of freedom.

## Appendix A2

Consider the additive risk model:

R(X_1_, X_2_,..., X_k_) = a_0 _+a_1_X_1_+ a_2_X_2_+...+ a_k_X_k_

where a_0 _= I and a_i _= (R_i_-1)I.

The average rate of the disease in the population at risk is given by

$$\text{A}=\sum_{{\text{X}}_{1},{\text{X}}_{2},...,{\text{X}}_{\text{k}}}\text{R}({\text{X}}_{1},{\text{X}}_{2},...,{\text{X}}_{\text{k}})\text{f}({\text{X}}_{1},{\text{X}}_{2},...,{\text{X}}_{\text{k}}),$$

where the probability density function, f, is given by (1).

It can be shown that A = a_0 _+a_1_G_1_+ a_2_G_2_+...+ a_k_G_k_.

Using the notations described for multiplicative models, the probability density function of the exposure levels of k genetic variants among cases is given by:

$$\text{g}({\text{U}}_{1},{\text{U}}_{2},...,{\text{U}}_{\text{k}})=({\text{a}}_{0}+\sum_{\text{i}=1}^{\text{k}}{\text{a}}_{\text{i}}{\text{U}}_{\text{i}})\prod_{\text{i}=1}^{\text{k}}{\text{G}}_{\text{i}}^{{\text{U}}_{\text{i}}}{\left(1-{\text{G}}_{\text{i}}\right)}^{\left(1-{\text{U}}_{\text{i}}\right)}/\text{A}$$

This is not an identifiable probability density function. Although it can be shown that the marginal distributions have asymptotically normal distributions, this does not guarantee the asymptotic normality of the joint distribution.
